# Virus-like Particles for TEM Regulation and Antitumor Therapy

**DOI:** 10.3390/jfb13040304

**Published:** 2022-12-17

**Authors:** Zhu Yang, Yongjie Chi, Jiaxin Bao, Xin Zhao, Jing Zhang, Lianyan Wang

**Affiliations:** 1 Key Laboratory of Green Process and Engineering, State Key Laboratory of Biochemical Engineering, Institute of Process Engineering, Chinese Academy of Sciences, Beijing 100190, China; 2 School of Chemical Engineering, University of Chinese Academy of Sciences, Beijing 100049, China; 3 School of Pharmacy, Heilongjiang University of Traditional Chinese Medicine, Harbin 150040, China

**Keywords:** virus-like particle, tumor microenvironment, immunotherapy, nanovaccine

## Abstract

Tumor development and metastasis are intimately associated with the tumor microenvironment (TME), and it is difficult for vector-restricted drugs to act on the TME for long-term cancer immunotherapy. Virus-like particles (VLPs) are nanocage structures self-assembled from nucleic acid free viral proteins. Most VLPs range from 20–200 nm in diameter and can naturally drain into lymph nodes to induce robust humoral immunity. As natural nucleic acid nanocarriers, their surfaces can also be genetically or chemically modified to achieve functions such as TME targeting. This review focuses on the design ideas of VLP as nanocarriers and the progress of their research in regulating TME.

## 1. Introduction

Cancer, a disease in which somatic cells accumulate mutations to achieve replicative immortality, invasive metastasis, and immune escape, is ranked as a leading death cause worldwide with more new cases being reported each year [1,2,3]. Nevertheless, cancer fatalities in the U.S. decreased by 32% as of 1991, and the age-standardized mortality rates declined by 1.2% per year in China [4,5]. This is attributed to early diagnosis and advances in innovative therapies, including improvement of traditional therapies and combination therapies with immunotherapy [6,7]. However, these applications are restrained by systemic toxicities, drug resistance, and dose dependence [8]. To address these concerns, nanomedicines have been applied extensively for tumor targeting [9], imaging monitoring [10], model building [11], etc. Through novel drug administration [12,13], innovate photothermal and other therapies [14]—combined with chemotherapy—nano anti-cancer agents are gradually coming to the clinic use. By targeting only specific cells—generally cancerous cells—while remaining unharmed to other cells and tissues, the engineered synthetic nanoparticles achieve beneficial biocompatibility, biosecurity, and bio-responsiveness and are a strategic approach to precision medicine compared to conventional clinical therapies [15,16,17,18].

From an evolutionary and eco-dynamic perspective, cancer is the consequence of a reciprocal dynamic balance between cancer cells and the tumor microenvironment (TME) [19]. The TME consists of non-tumor cells (including infiltrating cells, endothelial cells, fibroblasts), extracellular matrix (ECM), vasculature, and chemokines, etc. [20]. Like the ideology of the transformation of opposites in materialistic dialectics, the cells and cytokines in the TME can become friends or foes under the appropriate conditions. Tumor-associated macrophages (TAMs) can be repolarized into pro-inflammatory phenotypic M1-like macrophages under stimulation such as PI3Kγ inhibitors, TLR-7,8,9 agonists, TNF-α, and so on [21,22]. Tissue-resident anti-tumor γδ T cells promote tumor growth when infiltrated by tumor [23]. IL-17 produced by cells in adoptive T-cell treatment is anti-tumorigenic, but protumorigenic in mice deficient models [24]. The TME is the definite step for the validation of immune checkpoint inhibitors (ICI) and its regulators are promising targets for cancer immunotherapy [25,26].

**Figure 1:**
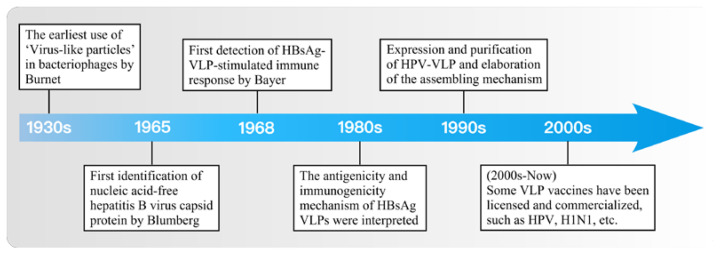
A brief timeline of major VLP studies.

Virus-like particles (VLPs), self-assembled viral proteins that can infect cells but lack of genetic material, are highly immunogenic and biologically active, which are morphologically and structurally similar to natural viral particles [27,28]. Since its discovery in the 1960s, VLP without nucleic acid has had a significant function as a new biological tool in the field of biomedical engineering [29,30] (Figure 1). VLPs can be classified into two structures: enveloped or capsid. Unlike non-enveloped VLPs comprised of capsid proteins, the former such as SARS coronavirus VLPs, consists of matrix proteins and glycoproteins contained in the lipid layer [31]. The exterior of VLPs facilitates genetic and chemical modifications to present different epitopes for functions such as in vivo long circulation and induction of humoral and cellular immunity [32,33]; the interior can carry proteins, nucleic acids, or compounds for specific types of intracellular delivery [34]. Compared to other nanoparticles, this makes VLPs a promising strategy for diversified development as therapeutic vaccines for different tumors (Table 1). In this review, we will focus on VLPs as flexible nanocarriers for TEM regulation and antitumor therapy.

## 2. Research and Development of VLP-Based Vaccine Design

VLPs consist of hundreds to thousands of protein molecules that display the major immunogenic region (MIR) in a rational and highly repetitive conformation, eliciting strong cell and humoral immunity [41]. They range in size from 10 nm to 1 µm and are mostly icosahedral or helical in structure, but of course also include spherical, rod and tubular forms, which are readily taken up by antigen presenting cells (APCs) and presented to T lymphocytes [42]. In addition, VLPs can bind to natural IgM or to C1q molecules, which are then deposited and taken up by follicular dendritic cells (FDCs) [36]. Thus, there are numerous cases of VLP-based immunotherapy in the clinical trials (Table 2).

**Figure 2:**
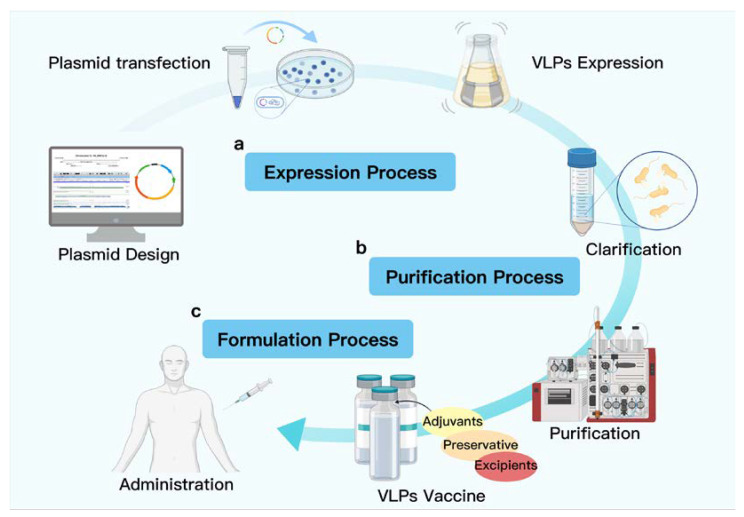
VLP-based vaccine production process: expression, purification, and formulation. (a) Production phase: including plasmid design for the construction of the target function and the selection of an appropriately viable expression platform to obtain self-assembling VLPs or monomers (E.coli expression examples are selected here); (b) Purification stage: using salting, ion exchange, ultracentrifugation, and other methods to obtain pure protein with host nucleic acid removed; (c) Formulation phase: add adjuvants, preservatives, excipients, and other ingredients to obtain sterile, safe, and efficient VLP vaccine products.

The production process for VLP-based vaccines generally consists of three main components: production, purification, and formulation [43]. VLPs can be produced by approximately 170 different host expression systems including bacteria, insects, yeast, and mammals, reflecting the large host spectrum of viruses from which VLPs are derived. After collecting and lysing the cells, they are clarified by centrifugation to remove cell debris and aggregates, further purified by ion exchange chromatography and finally polished to remove nucleic acids and endotoxins [44]. The final formulation process is completed by sterile processing and adjuvant formulation to produce a safe and efficient vaccine product (Figure 2).

### 2.1. Impact of Physical Attributes on VLP Delivery

Nanoparticle fluid dynamics in blood vessels is highly dependent on the size and geometry of the construct [45]. It is believed that nanoparticles smaller than 5 nm are rapidly cleared from the blood by the kidneys, while NPs larger than 200 nm accumulate in the liver and spleen [46]. For the vast majority of VLPs (20 to 200 nm), they can freely exit the lymphatic system by diffusing directly into the 200 nm pores of the lymphatic vessel walls. Mohsen et al. [47] found that Qβ-VLPs (20–30 nm) can accumulate in popliteal drainage LN as fast as 10 min after injection into the mouse footpad. The subcutaneous injection had similar results to the intravenous injection. Manolova et al. [48] used Alexa-488 labelled Qβ-VLPs (30 nm) injected into the footpads of C57BL/6 mice, compared to fluorescent polystyrene nanoparticles of 20, 500, and 1000 nm diameter. Two hours after injection, VLP and 20-nm beads had reached the popliteal LN and specifically targeted LN-resident cells. In contrast, the 500 and 1000 nm nanoparticles took 24 to 48 h to be delivered to the LN in a DC-dependent manner. Trafficking of VLPs can also be achieved by passive transfer in addition to free drainage. Ye et al. [49] found JC VLPs (53 nm) could enter brain endothelial cells and cross the BBB via clathrin-dependent mechanisms, and exocytosis or transcytosis of VLPs across the BBB was observed in vitro.

Geometry is an essential factor to consider in the design of nanovaccines, especially for VLPs that can self-assemble into higher order nanostructures. Viruses with the non-spherical appearance tend to evade recognition by the immune system and may have an advantage over spherical particles in terms of their prolonged in vivo circulation time [50]. For example, Tamminen et al. [51] found that different morphologies of rotavirus (RV) inner-capsid protein VP6 oligomers could be obtained by adjusting pH, and nonTS-VP6 (non-tubular/non-spherical VP6 assembly, 82.7 ± 35.1 nm) aggregate-like structures could be uptaken more than S-VP6 (spherical VP6, 114.6 ± 53.4 nm) by BMDCs, even when both are similar in size. Furthermore, T-VP6 (tubular VP6) is internalized and/or presented to the immune cells with greater efficacy than norovirus VLPs. Similarly, Zinkhan et al. [52] used C- or N-terminal insertion of a generic tetanus toxin (TT) epitope to specifically form CCMV-VLPs and found that Round-shaped CCMVTT-VLPs (~30 nm) induced IgG antibodies and reduced switching to IgG2b/IgG2c in comparison to the Round-shaped CCMVTT-VLPs. Although the mechanisms of how particle shape affects its behavior in vivo are not yet known, they will eventually be explained with increased attention and theoretical refinement.

### 2.2. Modifying the External Region of VLPs for Robust Humoral and Cellular Immunity

The target of therapeutic cancer vaccine is to reactivate the adaptive immune system against tumor-associated antigens (TAAs) to inhibit tumor growth. Therefore, the strategies for successful cancer vaccines include antigen presentation to DCs, DC activation, induction of CD4^+^ T cell and cytotoxic T lymphocyte (CTL) responses, and sustained regulation of TME. Correspondingly, to deliver a large number of target antigens to the APC in a high-quality and natural configuration, the exterior shell of the VLP can be modified using genetic engineering strategies or chemical coupling as detailed hereunder. This is easily achieved with VLP because of its high specific surface area and highly repetitive geometric appearance.

**Figure 3:**
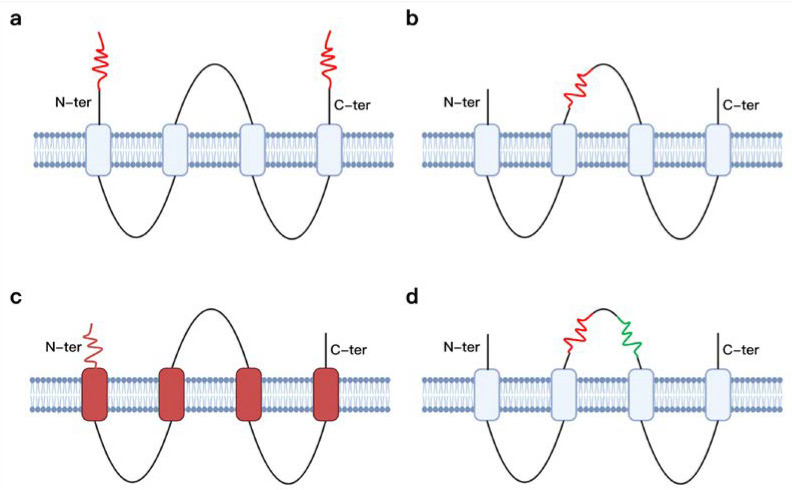
Sketch of commonly used VLP genetic engineering modification strategies (here multi-process membrane proteins are used as examples). (**a**) N/C-ter adjunction; (**b**) Exposed-loops insertion; (**c**) TM/CT grafting; (**d**) Multi-domain assembly.

Through genetic modification, exogenous peptides, epitopes, and even intact proteins can be easily integrated into chimeric VLPs. There are several methods according to the modification parts and objects: N/C-ter adjunction, exposed-loops insertion, TM/CT grafting [53], and multi-domain assembly (Figure 3). For capsid VLPs, which are also known as core protein VLPs, the most common strategy is to introduce exogenous peptides into the complete, truncated, or removed N-terminus/C-terminus of the capsid protein. Dishlers et al. [54] added the HBV preS1 sequence to the c-terminus of HBc-VLP to expose its epitope to the surface and demonstrated significant immunogenicity. The insertion of an exogenous PAMP epitopes into an exposed loop on the external surface is also an excellent example that can elicit a strong B-cell response without affecting monomer self-assembly. Hyakumura et al. [55] created hypoglycosylated HBsAgS-VLPs by introducing glycosylation sites in the outer loop region of HBsAgS to induce durable antibody responses than WT VLPs. Wei et al. [56] constructed an internal NP/external M2e bionic dual-antigen influenza vaccine by inserting influenza virus M2e (matrix protein 2 ectodomain) and NP (nucleoprotein) linear epitopes in the C-terminal MIR region of HBc, respectively. This vaccine provides complete prevention of H1N1 virus and stimulates a stronger germinal center (GC) B-cell and CD8^+^ T-cell response. For envelope VLP, foreign antigens are often incorporated into the transmembrane domain and cytoplasmic tail (TM/CT) of the transmembrane proteins in the viral envelope. Cai et al. [57] constructed a chimeric preS VLP where the signal peptide and TM/CT of influenza virus HA protein were fused to the C- and N-terminus of preS sequence, respectively, and where PreS VLP could trigger robust and specific humoral immunity. In addition to introducing antigenic sequences at various positions, different structural domains can also be linked and aggregated into multifunctional VLPs. This includes the assembly of two monomeric MIR regions by linker tandem fusion as a single polypeptide chain [58], and also includes the exchange of different structural domains of the same coat protein for their respective self-assembly [59].

The target peptide or polysaccharide antigen can also be cross-linked to the surface of the VLP by chemical coupling, which focuses on the following residues: amino, sulfhydryl, carboxyl, etc., and their derivatives. Unnatural amino groups introduced after translation can be modified by click chemistry [60]. The double orthogonal, highly specific reaction between the azide and the alkyne moiety allows VLP to be modified by various antibodies [61,62]. Sortase covalently link proteins with N-terminal oligoglycine motifs to C-terminal LPXTGX motifs and have been proven to induce specific IgG responses [63]. For the modification of cysteine residues, it is more commonly formed by electrostatic interactions for electrostatic interaction locks (EILs) to interact to generate disulfide bonds. Xu et al. [64] achieved homogeneous in vitro self-assembly of SV40 in this way. Catcher/Tag technology allows the formation of spontaneous intramolecular homopeptide bonds between proteins to link foreign peptides and proteins to VLP. Sander et al. [65] attached P. falciparum Pfs48/45 protein to the surface of Acinetobacter phage AP205 VLP by SpyTag/SpyCatcher technology to achieve a stronger antibody response.

Although there are still many non-covalent bond modifications of VLP, such as His-Tag/Ni-NTA affinity and biotin-affinity [66], the non-covalent reaction with antigenic proteins is less stable than covalent response for carriers targeting TME and tumor therapy, and does not facilitate quantitative analysis and response release, so it will not be discussed here.

### 2.3. Packing the Interior Region of VLPs for Effective Adjuvant Effect

Vaccines containing only antigens do not usually build the desired immune response, and studies have found that adjuvant mechanisms such as PAMP, DAMP, metabolism, and epigenetics can activate innate immunity and maintain constant adaptive immunity [67,68]. Likewise to natural viruses, VLPs generally encapsulates host nucleic acids during self-assembly into nanocage structures. If VLP is disassembled and then sheared and removed from the natural nucleic acid using nucleases, its positively charged inner surface can be modified with various adjuvants through their pores or during the reassembly process, such as ssRNA, dsRNA, and CpGs motifs [47,69]. ssRNA can be encapsulated by VLP to be free from RNase enzymes degradation and to achieve TLR7/8 activation. Savelyeva et al. [70] designed plant virus particles (PVP) coupled to weak idiotypic(Id) tumor antigen and loaded with ssRNA induced a stronger antibody response than the Id vaccine. dsRNA or poly I:C induces type I interferon (IFN) production via TLR3-dependent MyD88 signaling pathway [71]. In contrast, the CpG of TLR9 ligands had shown markedly higher cell mediated immunity (CMI) [72].

## 3. VLPs for TEM Regulation against Cancer

For a reasonably effective tumor vaccine, it is important to achieve not only tumor prevention, but also ring-breaking, killing, and clearance of tumor cells. Thus, the key is to generate a cell-mediated immune response, especially a Th1 immune response. VLPs could be an excellent candidate for cancer vaccine development by activating the MHC-I pathway through delivering antigen to the cytoplasm to achieve these functions. It is an opportunity and challenge for targeted TME research to mature DC to activate adaptive immunity and relieve immunosuppressive signals in TME.

### 3.1. Targeting Dendritic Cells

Tumor-infiltrating DCs, as the first step in the anti-tumor immune response, tend to exhibit quantitative and functional defects in TME due to oncometabolites and tumor-derived suppressors [73,74]. To regulate their specific activation and maturation, DCs are usually targeted with VLPs that are co-loaded with antigens and coupled to specific ligands, like CD40, CD11c, CD205, or mannose receptor. Alam et al. [75] biocoupled different aryl mannose to Qβ-VLP to enable uptake via DC-SIGN. The results showed that Qβ-Man was only selectively taken up by DC-SIGN expressing cell lines and efficiently delivered to the endosomal compartments for DC maturation and expression of pro-inflammatory cytokines such as IL-1β. Miraculously, targeting different subpopulations of DC cells may also produce different therapeutic effects. Li et al. [76] found that recombinant VLP-gp33r was more effective than conjugated VLP-gp33c when used as a therapeutic vaccine because the former affected the induction of cytotoxic effector cells via Langerin^+^ DC. Incidentally, in addition to specifically targeting DC cells, some studies have looked at DC activation and maturation through the encapsulation of DC activating molecules by VLPs. Gomes et al. [77] fabricated the Qβ-E7-Cpg to ensure that DCs were properly activated through dual stimulation. Alternatively, vaccine efficacy can be increased by altering the delivery strategy, for example by intradermal administration through gene gun, laser treatment after intradermal injection, and electroporation after intramuscular injection [78]. Guo et al. [79] prepared microneedle patches containing OVA-HBc VLPs and mesoporous silica nanoparticles that could achieve 42% BMDC maturation in vitro to induce CD8^+^ T cell-mediated immune responses. With the characteristics of DCs, the delivery of antigens by VLPs has become the most fundamental and widespread design strategy. Specifically targeting DCs is effective in avoiding systemic toxicity or autoimmunity of nano-agents, but also therefore requires accurate antigens and appropriate dosage. Meanwhile, the functions of different DC subpopulations need to be studied in more depth to achieve the most appropriate formulation.

### 3.2. Targeting Tumor-Associated Macrophages

While most TAMs originate from monocytic precursors, recent studies have shown that tissue-resident macrophages (TRMs) originate from embryonic precursors and may maintain TAM levels [80]. As typical plastic cells, TAMs can undergo various forms of phenotypic polarization upon stimulation by different microenvironmental signals. From the perspective of cancer vaccine design, TAM are often simply divided into classically activated or inflammatory (M1) and alternatively activated or anti-inflammatory (M2) macrophages, depending on the expression of cell surface markers and their biological function. In response to hypoxia and changes in cytokines such as IL-4 in the TME, TAM1 repolarises to TAM2 to promote immunosuppression. For example, immune checkpoint ligands with elevated levels of TAM expression, such as PD-L1, PD-L2, etc., thereby directly inhibit T-cell activity [81].

Further, the utilization of VLPs to specifically deliver drugs to TAM and modulate phenotype reversal is a viable idea for cancer vaccines. For example, CpG-ODNs can promote M1 polarization in macrophages via TLR9. Cai et al. [82] used the disassembly and reassembly of CCMV to package ODN1826, which could significantly improve drug efficiency. Recombinant VLP is preferentially taken up by TAM and promotes M1 polarization, thereby enhancing the therapeutic effect on colon cancer and melanoma in mice. Given their small size and ability to cross physiological barriers, VLPs enable multiple modes of delivery to modulate the TME. Zhang et al. [83] loaded antigen and adjuvant via OVA-HBc-Poly (I:C) while compounding immunomodulator-containing (JQ1) liposomes for endotracheal administration in the lung. The results showed that the nanovaccine promoted M1 polarization, significantly reduced PD-L1 expression levels in the tumor-bearing lung, enhanced CTL response, and reshaped the tumor microenvironment. For clinical applications, the most practical approach remains to explore therapeutic strategies in combination with conventional therapies to achieve tumor suppression. An example is tumor regression after radiotherapy which can recruit inflammatory cells and can be followed by the addition of CMP-001 (Qβ-CpG) to activate a sustained anti-tumor effect [84].

Strategies to target TAM also include small molecule drugs such as Bruton’s tyrosine kinase (BTK) to inhibit TAM survival and function, but unfortunately there has not been more in-depth research in VLPs vectors [85]. Similarly, TAM of different origins, such as systemically recruited or tissue-resident, have different treatment outcomes. VLPs still have substantial unexplored advantages in targeting TAMs, such as the fusion of protein-like receptors such as M2-targeting peptides (M2pep) to VLPs for expression, or the loading of VLPs with other small molecule drugs. Ultimately, it is hoped that with a better understanding of TAMs and VLPs, nanovaccines will provide more ideas for tumor immunotherapy.

### 3.3. Targeting Tumor-Infiltrating Treg Cells

Although a variety of immunosuppressive T cells have been identified and studied, such as CD4^+^ type 1 T regulatory (Tr1) cells [86], the vast majority in the tumor microenvironment are still Treg cells (CD4^+^ CD25^+^ Foxp3^+^). Treg cells affect the normal work of responding T cells by binding with high affinity to IL-2 in the environment, while high expression of IL-10 and CTLA-4 inhibits CD80/CD86 expression in APCs and thus indirectly inhibits T cell co-stimulatory activation. Therefore cancer immunotherapy targeting Treg cells should choose molecules that are relatively specific to Treg depletion or functional regulation, such as CTLA-4, PD-1, OX-40, etc. [87]. Agonistic antibodies that antagonize Treg-mediated immunosuppression and activate T-cell proliferation can be selected to modify VLP. For example, Palameta et al. [88] generated 4-1BBL + OX40L bivalent VLP that significantly reduced the transformation of FoxP3-positive cells and increased T-cell proliferation and IFN-γ secretion. The PSMA ligand is then simultaneously attached for tumor cell targeting and anchored to GM-CSF for tumor targeting and stimulation of DCs differentiation. Another idea is to use checkpoint blocking antibodies that deplete the effect of Treg. Whether coupled to a PD-1 antibody or in combination therapy, the VLP vector usually requires T cell stimulator modification to better stimulate CD8^+^ T activation. Simons et al. [89] modified VLP with prostate cancer-associated tumor antigens and T-cell stimulators, and both the vaccine alone and the anti-PD1 antibody combination significantly reduced tumor load. Targeted Treg immunotherapy is usually accompanied by autoimmune side effects. Therefore, whether VLP or other carriers are used, the effector Treg in tumor tissue should be selectively targeted, and the dose and time should be adjusted to achieve the balance between tumor immunity and autoimmune.

Of course, there are also some studies targeting other TME components with VLP as a carrier, such as vascular venation [90]. However, due to the lack of systematic research, it is not discussed here.

## 4. Conclusions and Perspectives

With the rapid development of nanotechnology and protein engineering, the design of cancer vaccines based on VLPs has been greatly developed in recent years due to their homogeneous and stable structure, large number of functional structures expressed repeatedly, excellent biocompatibility, and the easy modification of the exposure sites. Numerous studies have shown that both external gene fusions and chemical modifications as well as internal drug loading need to start with the physicochemical properties of the VLP with the target in mind. The impact of its size, shape, isoelectric point, and other properties on pharmacokinetics distribution in the organism should be fully considered. In terms of external surface modifications, gene fusions allow for mass and accurate expression, providing enhanced targeting and immunological effects, but may require additional optimization in terms of vector preparation. Chemical coupling, on the other hand, requires additional consideration of the toxicity issues associated with the reaction conditions while maintaining the loading rate. The choice of internal adjuvants is another feature, which can achieve more powerful therapeutic effects than free drugs.

One of the challenges in VLP vaccine development is the expensive and complex production and purification process. Compared with other synthetic nanocarriers such as PLGA and liposomes, the production cycle is longer, which requires expression and correct self-assembly in host cells. To improve its biosafety, additional host nucleic acid and lipopolysaccharide endotoxin removal is required, which undoubtedly adds significantly to the time and economic cost of VLP vaccine production. Cell-free systems have been developed for this purpose [91], which greatly simplify the production steps, and in the future more simple and efficient purification of VLP will remain a priority for research. In addition, the fluid dynamics of the VLP vaccine in the blood still requires multidisciplinary studies, and its achievement of prolonged circulation within the blood and targeting of specific cells in the tumor microenvironment remain major challenges. Currently, PEGylation continues to be a viable option, and studies have been performed to protect VLP from immune responses through PEG modification [32,92].

Tumor vaccines have evolved with the understanding of the tumor microenvironment and the development of immunology, and the more the former is studied, the better the efficacy of nanomedicines, including VLP, can be achieved. For now, VLPs can be targeted to solid tumors and can also be used as preventive or therapeutic cancer vaccines in combination with conventional therapies and checkpoint inhibitors.

## Figures and Tables

**Table 1:** The pros and cons of virus-like particles compared with nanomaterials strategies.

Platforms	Pros	Cons	Ref.
Virus-Like Particles	(i) Highly ordered structures with stability at the nanoscale. (ii) Uniform size and shape distribution through self-assembly. (iii) Three distinct interfaces available for functionalization (external, internal, and inter-subunit). (iv) Both genetic and chemical modifications are available. (v) Repeatable structure means that a single modification allows the whole particle to be arranged in a constant manner.	(i) Viruses with mutant epitopes do not elicit an effective immune response. (ii) High immunity is easily eliminated by triggering an immune response. (iii) The purification step is complex.	[35,36,37]
Lipid-based nanoparticles	(i) Strong membrane fusion capability. (ii) Broad adaptability to the drugs contained.	(i) Poor or no immunogenicity. (ii) Toxicity (cytotoxicity and genotoxicity). (iii) The use of organic solvents in production may be detrimental to large-scale production.	[38,39,40,12]
Polymer nanoparticles	(i) Simple and scalable synthesis. (ii) High transfection rate. (iii) Prepared with widespread and accessible natural polymers.		
Inorganic nanoparticles	(i) Simple to synthesize. (ii) Excellent magnetic, optical, and electrical properties (ideal materials for building integrated diagnosis and therapy).		

**Table 2:** Key Clinical Trials of VLP-Based for Immune related diseases.

Study	Conditions	Interventions	Phases	Completion Date	NCT Number
A Study to Compare Immune Response of V503 to Gardasil in 16- to 26-year-old Men (V503-020)	Papilloma Viral Infection	V503 vs. GARDASIL	Phase 3	22 April 2015	NCT02114385
Trial of a Chikungunya Vaccine, PXVX0317 CHIKV-VLP, in Healthy Adults	Chikungunya Virus Infection	CHIKV VLP/unadjuvanted vs. CHIKV VLP/adjuvanted vs. Placebo	Phase 2	21 September 2020	NCT03483961
Serologic Assay Validation, Proficiency Testing, Safety, and Immunogenicity of Norovirus GI.1/GII.4 Bivalent Virus-Like Particle Vaccine	Norovirus, Healthy Participants	NoV GI.1/GII.4 Bivalent VLP Vaccine	Phase 2	9 September 2015	NCT02475278
A Study of V503 (A Multivalent Human Papillomavirus [HPV] L1 Virus-Like Particle [VLP] Vaccine) in Preadolescents and Adolescents (V503-002)	Cervical Cancers, Vulvar Cancer, Vaginal Cancer, Genital Lesions, PAP Test Abnormalities, HPV Infections	V503	Phase 3	22 April 2021	NCT00943722
VRC 313: A Trivalent Virus-like Particle (VLP) Encephalitis Vaccine (WEVEE) in Healthy Adults	Venezuelan Equine Encephalitis, Western Equine Encephalitis, Eastern Equine Encephalitis, Alphavirus Infections	VRC-WEVVLP073-00-VP vs. VRC-GENMIX083-AL-VP	Phase 1	26 February 2020	NCT03879603
Safety and Immunogenicity of GSK Biologicals’ HPV-16/18 L1 VLP AS04 Vaccine (GSK-580299) in Healthy Female Children 4–6 Years Old	Infections, Papillomavirus	Cervarix vs. Priorix vs. Infanrix	Phase 3	6 October 2016	NCT01627561
A Study of Gardasil (V501) in Preadolescents and Adolescents (V501-018)	Human, Papillomavirus Infections	V501 vs. Placebo	Phase 3	1 June 2015	NCT00092547
Trial for Safety and Immunogenicity of a Chikungunya Vaccine, VRC-CHKVLP059-00-VP, in Healthy Adults	Chikungunya Virus Infection	VRC-CHKVLP059-00-VP vs. VRC-PBSPLA043-00-VP	Phase 2	6 March 2018	NCT02562482
Gardasil Vaccination in Post Stem Cell Transplant Patients	Gardasil Vaccine, Stem Cell Transplant, Immunogenicity	Gardasil	Phase 1	19 July 2016	NCT01092195
Safety and Immunogenicity of Norovirus Bivalent Virus-Like Particle Vaccine in Healthy Adults	Norovirus Prevention	Norovirus Bivalent VLP Vaccine vs. Placebo (Saline)	Phase 2	6 January 2016	NCT02142504
Safety and Immunogenicity of Norovirus GI.1/GII.4 Bivalent VLP Vaccine	Healthy Volunteers, Norovirus, Prevention	Hepatitis A Vaccine vs. Norovirus Bivalent VLP Vaccine	Phase 2	19 June 2015	NCT02038907
Safety and Immunogenicity of Norovirus GI.1/GII.4 Bivalent Virus-Like Particle Vaccine in an Elderly Population	Norovirus	Norovirus GI.1/GII.4 Bivalent VLP Vaccine vs. 0.9% sodium chloride (saline)	Phase 2	29 September 2017	NCT02661490

## Data Availability

Not applicable.
